# Expression of adiponectin receptors 1 (AdipoR1) and 2 (AdipoR2) in the porcine pituitary during the oestrous cycle

**DOI:** 10.1186/1477-7827-11-18

**Published:** 2013-03-05

**Authors:** Marta Kiezun, Anna Maleszka, Nina Smolinska, Anna Nitkiewicz, Tadeusz Kaminski

**Affiliations:** 1 Department of Animal Physiology, University of Warmia and Mazury in Olsztyn, Oczapowskiego 1A, Olsztyn-Kortowo, 10-710, Poland

**Keywords:** Adiponectin, Adiponectin receptor, Pituitary, Oestrous cycle, Pig

## Abstract

**Background:**

Adiponectin, protein secreted mainly by white adipose tissue, is an important factor linking the regulation of metabolic homeostasis and reproductive processes. The biological activity of the hormone is mediated *via* two distinct receptors, termed adiponectin receptor 1(AdipoR1) and adiponectin receptor 2 (AdipoR2). The present study analyzed mRNA and protein expression of AdipoR1 and AdipoR2 in the anterior (AP) and posterior (NP) pituitary of cyclic pigs.

**Methods:**

The total of 20 animals was assigned to one of four experimental groups (n = 5 per group) as follows: days 2–3 (early-luteal phase), 10–12 (mid-luteal phase), 14–16 (late-luteal phase), 17–19 (follicular phase) of the oestrous cycle. mRNA and protein expression were analyzed using real-time PCR and Western Blot methods, respectively.

**Results:**

The lowest AdipoR1 gene expression was detected in AP on days 10–12 relative to days 2–3 and 14–16 (p < 0.05). In NP, AdipoR1 mRNA levels were elevated on days 10–12 and 14–16 (p < 0.05). AdipoR2 gene expression in AP was the lowest on days 10–12, and an expression peak occurred on days 2–3 (p < 0.05). In NP, the lowest (p < 0.05) expression of AdipoR2 mRNA was noted on days 17–19. The AdipoR1 protein content in AP was the lowest on days 17–19 (p < 0.05), while in NP the variations in protein expression levels during the oestrous cycle were negligible. AdipoR2 protein in AP was most abundant on days 10–12, and it reached the lowest level on days 2–3 and 17–19 of the cycle (p < 0.05). The presence of AdipoR2 protein in NP was more pronounced on days 10–12 (p < 0.05).

**Conclusions:**

Our study was the first experiment to demonstrate that AdipoR1 and AdipoR2 mRNAs and proteins are present in the porcine pituitary and that adiponectin receptors expression is dependent on endocrine status of the animals.

## Background

Adiponectin, also termed Acrp30, apM1, GBP28 and AdipoQ, was first identified by four independent research groups [1-4]. This 30-kDa adipose tissue-derived hormone is highly abundant in human and mouse serum and shows an inverse correlation with the body mass index [5,6]. Adiponectin monomers have an amino-terminal collagen-like domain and a carboxy-terminal globular domain that generate trimers, hexamers and high-molecular-weight (HMW) multimers. Several studies have demonstrated that different multimeric forms can determine the activity of adiponectin [7-9]. The discussed protein acts *via* AMP-activated protein kinase (AMPK) to modulate glucose and lipid metabolism [10]. It is also known for its protective role in obesity-related disorders, such as insulin resistance, type 2 diabetes mellitus and atherosclerosis [6,11], as well as in carcinogenesis [12]. A beneficial effect of adiponectin on female reproductive function was also suggested [13].

Two distinct adiponectin receptors (AdipoR1, AdipoR2) are seven-transmembrane domain receptors with an extracellular carboxyl terminus and an intracellular amino terminus, thus they are opposite to the topology of other G-coupled protein receptors. AdipoR1 shows high-affinity for the globular form of adiponectin, and AdipoR2 has an intermediate affinity for both full-length and globular species. Both adiponectin receptors are highly related and share 66.7% sequence identity in mice [14]. AdipoR1 and AdipoR2 are widely expressed, which suggests that adiponectin has pleiotropic effects. The highest expression of type 1 adiponectin receptor is noted in human, murine, porcine and chicken skeletal muscles, and the highest expression of type 2 adiponectin receptor is observed in the liver of the above forms [14-16]. The third putative adiponectin receptor, T-cadherin (cadherin 13, CDH13), is a molecule that lacks the transmembrane and cytoplasmatic domains and is bound to the surface membrane through a glycosylphosphatidylinositol anchor. The expression of T-cadherin was observed to confer binding of hexameric and HMW multimers but not trimeric adiponectin. It is also postulated that it may compete with AdipoRs for adiponectin binding or interfere with adiponectin signal transduction [17].

It has long been recognized that reproductive function is closely associated with energy balance, and metabolic dysregulation is linked with reproductive abnormalities. Neumeier et al. [18] reported that adiponectin is able to cross the blood-brain barrier, also subsequent studies showed that both AdipoRs and adiponectin are expressed in the human and rat brain and pituitary [19-21]. Adiponectin seems to be involved in autocrine/paracrine control of pituitary somatotrophs and gonadotrophs and secretion of GH, LH and FSH [19,21]. Thus, one can assume that adiponectin affects secretion of ovarian steroid hormones. However, there is lack of data describing the inverse relationship, i.e. the influence of hormonal status of animals, typical for each phase of the oestrous cycle, on pituitary adiponectin system expression. To our knowledge, no research has investigated the expression of the adiponectin receptors in the anterior (AP) and posterior (NP) pituitary of pigs during the cycle. The aim of this study was to compare the expression of AdipoR1 and AdipoR2 in the AP and NP of pigs at four stages of the oestrous cycle with the use of real-time PCR and Western blotting techniques.

## Methods

### Experimental animals and tissue collection

The studies were carried out in accordance with the principles and the procedures of the Animal Ethics Committee at the University of Warmia and Mazury in Olsztyn. Mature gilts (Large White × Polish Landrace) at 7–8 months of age, with body weight of 120–130 kg, descended from private breeding, were used. The total of 20 animals was assigned to one of four experimental groups (n = 5 per group) as follows: days 2–3 (early-luteal phase), 10–12 (mid-luteal phase), 14–16 (late-luteal phase), 17–19 (follicular phase) of the oestrous cycle. Females were monitored daily for oestrus behaviour in the presence of an intact boar. The onset of the second oestrus was marked as day 0 of the oestrous cycle. Phase of the oestrous cycle was also confirmed on the basis of morphology of the ovary [22]. To confirm correctness of the evaluation of the oestrous cycle phase, the level of progesterone (P_4_) was determined as described by Nitkiewicz et al. [23]. The plasma level of P_4_ on days 2–3, 10–12, 14–16, and 17–19 was as follows: 4 ± 2 ng/ml, 19 ± 3.4 ng/ml, 8 ± 2.2 ng/ml, and 0.2 ± 0.03 ng/ml, respectively, and corresponds with earlier published data pertaining to the steroid concentration in pig plasma during the oestrous cycle [24]. Within a few minutes after slaughter the pituitary gland, muscle and liver were removed. Next, pituitary gland was separated into anterior and posterior lobes. All of the samples were frozen in liquid nitrogen and stored at −80°C until processing for RNA and protein analysis. The same samples of tissues were used for RNA and protein isolation.

### Total RNA isolation and cDNA synthesis

Total RNA was extracted from all collected tissues using the Absolutely RNA Miniprep Kit (Stratagene, USA). RNA concentration and quality were determined spectrophotometrically (NanoDrop ND-1000, NanoDrop Technologies Inc., USA). The entire total RNA was intact with high quality, i.e. optical density (O.D.) 260/280 and 260/230 ratios were between 1.8 and 2.0 and 1.8 or greater, respectively. Approximately 1 μg of RNA was reverse transcribed into cDNA in a total volume of 20 μl with 0.5 μg oligo (dT)_15_ primer (Roche, Germany) using Omniscript RT Kit (Qiagen, USA) at 37°C for 1 h and was terminated by incubation at 93°C for 5 min.

#### 2.3 Quantitative real-time PCR

Quantitative real-time PCR analysis was performed using a PCR System 7300 (Applied Biosystems, USA) with SYBR Green. Forward and reverse primers were selected according to Lord et al. [15]: AdipoR1, forward: 5^′^-GCCATGGAGAAGATGGAGGA-3^′^, reverse: 5^′^-AGCACGTCGTACGGGATGA-3^′^; AdipoR2, forward: 5^′^-TGTTCGCCACCCCTCAGTAT-3^′^, reverse: 5^′^-AATGATTCCACTCAGGCCCA-3^′^; cyclophilin A, forward: 5^′^-GCACTGGTGGCAAGTCCAT-3^′^, reverse: 5^′^-AGGACCCGTATGCTTCAGGA-3^′^. AdipoR1 primers (access no: AY452710) were complementary to positions 148–168 (F) and 204–222 (R) of pig AdipoR1 gene sequence, AdipoR2 primers (access no: AY452711) were complementary to positions 322–341 (F) and 373–392 (R) of pig AdipoR2 gene sequence, cyclophilin A primers (access no: AY266299) were complementary to positions 219–237 (F) and 269–299 (R) of porcine cyclophilin A gene sequence. The constitutively expressed gene, cyclophilin A, was used as the internal control to verify the quantitative real-time PCR. During the preliminary experiments it was found that expression of cyclophilin A mRNA was very similar in both lobes of the pituitary and was stable during the oestrous cycle. The PCR reaction included 20 ng cDNA, 300 nM (AdipoR1 forward, cyclophilin A forward and reverse), 50 nM (AdipoR1 reverse, AdipoR2 forward and reverse) primers, 12.5 μl SYBR Green PCR Master Mix (Applied Biosystems, USA), and RNase free water in a final volume of 25 μl. Quantitative real-time PCR cycling conditions were as follows: 50°C for 2 min, then 95°C for 10 min for initial denaturation and enzyme activation, followed by 40 cycles of denaturation at 95°C for 15 s, and annealing at 60°C for 1 min. Negative controls were performed using water as a substitute for cDNA, or reverse transcription was not performed prior to PCR. All samples were amplified in duplicate. The specificity of amplification was tested at the end of the PCR by melting-curve analysis. Product purity was confirmed by electrophoresis. Calculation of relative expression levels of AdipoR1 and AdipoR2 was conducted based on the comparative cycle threshold method (ΔΔC_T_) [25]. Expression of AdipoR1 and AdipoR2 was calculated by the equation 2 ^–ΔΔCT^, where ΔC_T_ was obtained by subtracting the corresponding cyclophilin A C_T_ value from specific C_T_ of the target (AdipoR1 or AdipoR2, and ΔΔC_T_ was determined by subtracting the ΔC_T_ of each experimental sample from ΔC_T_ of the reference sample, called the calibrator (the tissue with the lowest expression).

### Western blotting

Western blotting analysis was performed essentially as described by Smolinska et al. [26]. Briefly, equal amounts of porcine pituitary lysates (anterior and posterior parts separately, 10 μg of total proteins) were resolved by SDS-PAGE (12.5%) for separating AdipoR1, AdipoR2 and actin and transferred to nitrocellulose membranes (Whatman, USA). Blots were blocked for 5 h at 4°C in Tris-buffered saline Tween-20 containing 5% skimmed milk powder, then incubated overnight at 4°C with the rabbit polyclonal adiponectin receptor 1 antibodies at a dilution of 1:150 (Phoenix Pharmaceuticals, USA), rabbit polyclonal adiponectin receptor 2 antibodies (Phoenix Pharmaceuticals, USA) at a dilution of 1:200, or rabbit polyclonal actin antibodies (Sigma, USA) diluted 1:200, which were used as an internal control for equal loading as well as to quantify porcine AdipoR1 and AdipoR2 proteins. To identify immunoreactive bands, membranes were incubated for 1.5 h at room temperature with mouse anti-rabbit IgG for AdipoR1 (Sigma, USA; diluted 1:2000), goat anti-rabbit IgG for AdipoR2 (Santa Cruz Biotechnology, USA; diluted 1:500) or goat anti-rabbit IgG for actin conjugated with alkaline phosphatase (Santa Cruz Biotechnology, USA; diluted 1:5000). Nonspecific foetal calf serum (MP Biomedicals, USA) was used instead of primary antibodies to produce negative control blots. The immunocomplexes were visualized using 4-nitroblue tetrazolium chloride (NBT) and 5-bromo-4-chloro-3-indolyl phosphate (BCIP), according to the manufacture’s protocol (Promega, USA). The same procedures were used for preparing positive controls-skeletal muscle and liver (for AdipoR1 and AdipoR2, respectively). The results of Western blotting were quantified by densitometric scanning of immunoblots with GelScan for Windows ver. 1.45 software (Kucharczyk, Poland). Data were expressed as ratio of AdipoR1 or AdipoR2 protein relative to actin protein in arbitrary optical density units.

### Data analysis

Data are presented as means ± S.E.M. from five different observations. Differences between groups were analysed by two-way ANOVA followed by least significant differences (LSD) post-hoc test. Statistical analyses were performed using Statistica Software (StatSoft Inc., Tulsa, USA). Values for *p* < 0.05 were considered statistically significant.

## Results

### Quantitative real-time PCR

**Figure 1:**
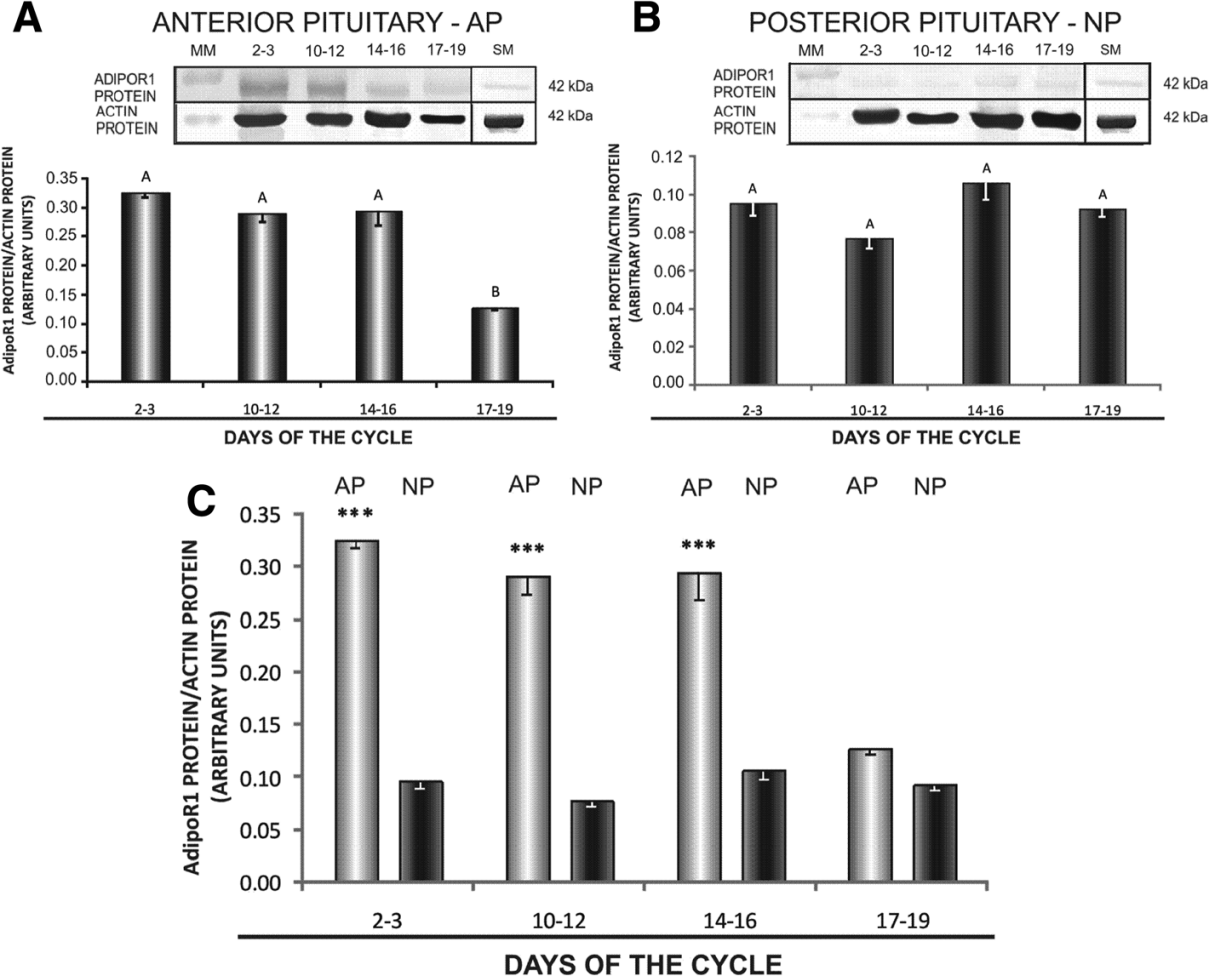
**Expression of AdipoR1 mRNA in the anterior and posterior pituitary.** Comparison of adiponectin receptor 1 (AdipoR1) mRNA expression determined by quantitative real-time PCR in porcine anterior (**A**) and posterior (**B**) pituitary glands between days 2–3, 10–12, 14–16 and 17–19 of the oestrous cycle, and (**C**) between anterior and posterior pituitary glands on days 2–3, 10–12, 14–16 and 17–19 of the cycle. Results are means ± S.E.M. (n = 5). Bars with different superscripts are significantly different. Capital letters indicate *p* < 0.05; **p < 0.01; ***p < 0.001.

**Figure 2:**
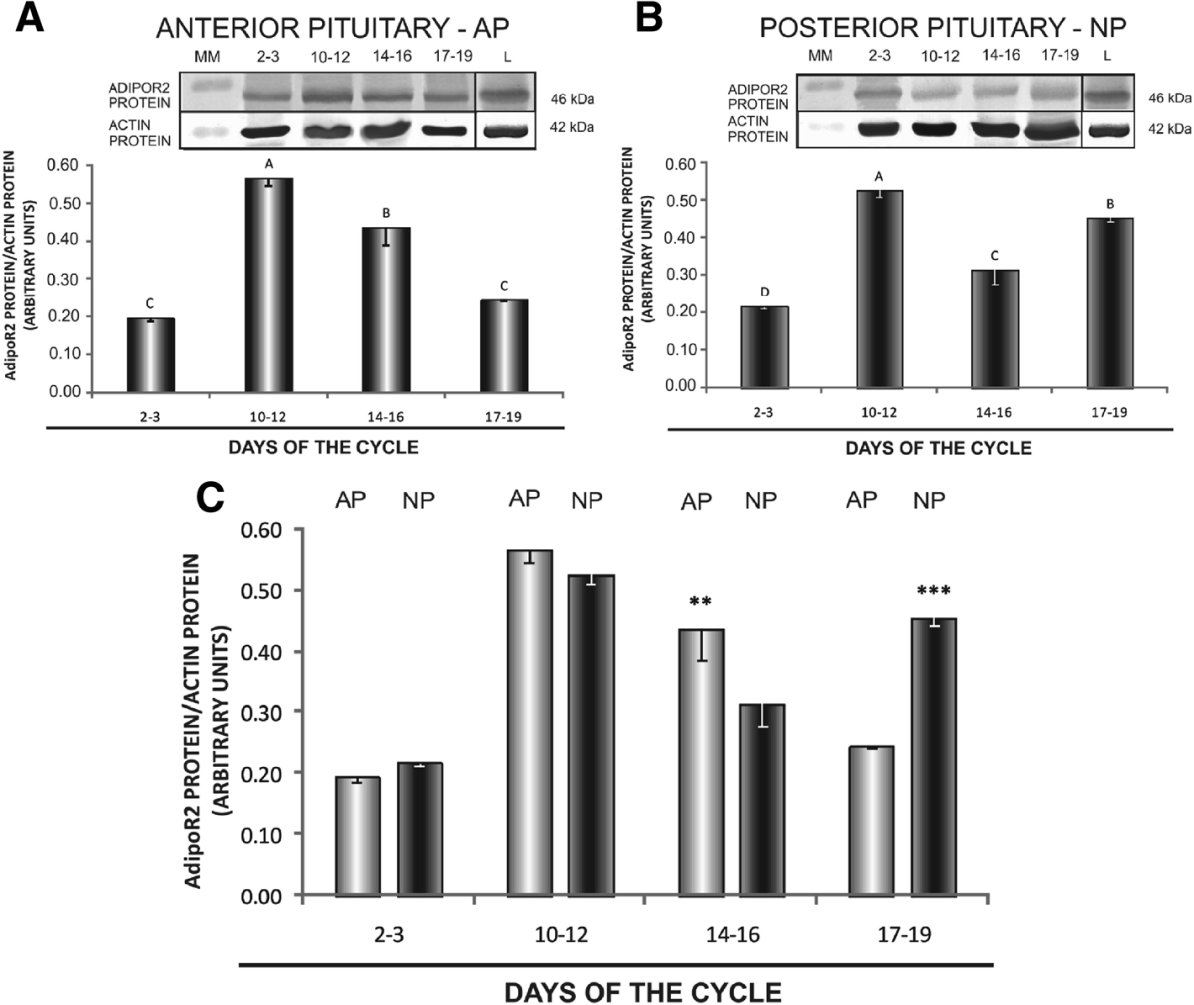
**Expression of AdipoR2 mRNA in the anterior and posterior pituitary.** Comparison of adiponectin receptor 2 (AdipoR2) mRNA expression determined by quantitative real-time PCR in porcine anterior (**A**) and posterior (**B**) pituitary glands between days 2–3, 10–12, 14–16 and 17–19 of the oestrous cycle, and (**C**) between anterior and posterior pituitary glands on days 2–3, 10–12, 14–16 and 17–19 of the cycle. Results are means ± S.E.M. (n = 5). Bars with different superscripts are significantly different. Capital letters indicate *p* < 0.05;**p < 0.01; ***p < 0.001.

In AP, the lowest AdipoR1 mRNA levels were reported on days 10–12 (*p* < 0.05) compared to days 2–3 and 14–16, whereas in NP, higher levels of AdipoR1 mRNA were noted on days 10–12 and 14–16 (*p* < 0.05) (Figure 1A and B). AdipoR2 gene expression in AP was the highest on days 2–3 (*p* < 0.05 in relation to days 10–12 and 14–16), and it was the lowest on days 10–12 of the oestrous cycle (*p* < 0.05) (Figure 2A). The level of AdipoR2 mRNA in NP was markedly lower on days 17–19 (*p* < 0.05 relative to the remaining periods of the cycle) (Figure 2B). mRNA transcripts for both AdipoR1 and AdipoR2 were higher in NP than in AP on days 10–12 and 14–16 (*p* < 0.05); the differences between AP and NP were not significant during the remaining stages of the oestrous cycle (Figures 1C and 2C).

### Western blotting

**Figure 3:**
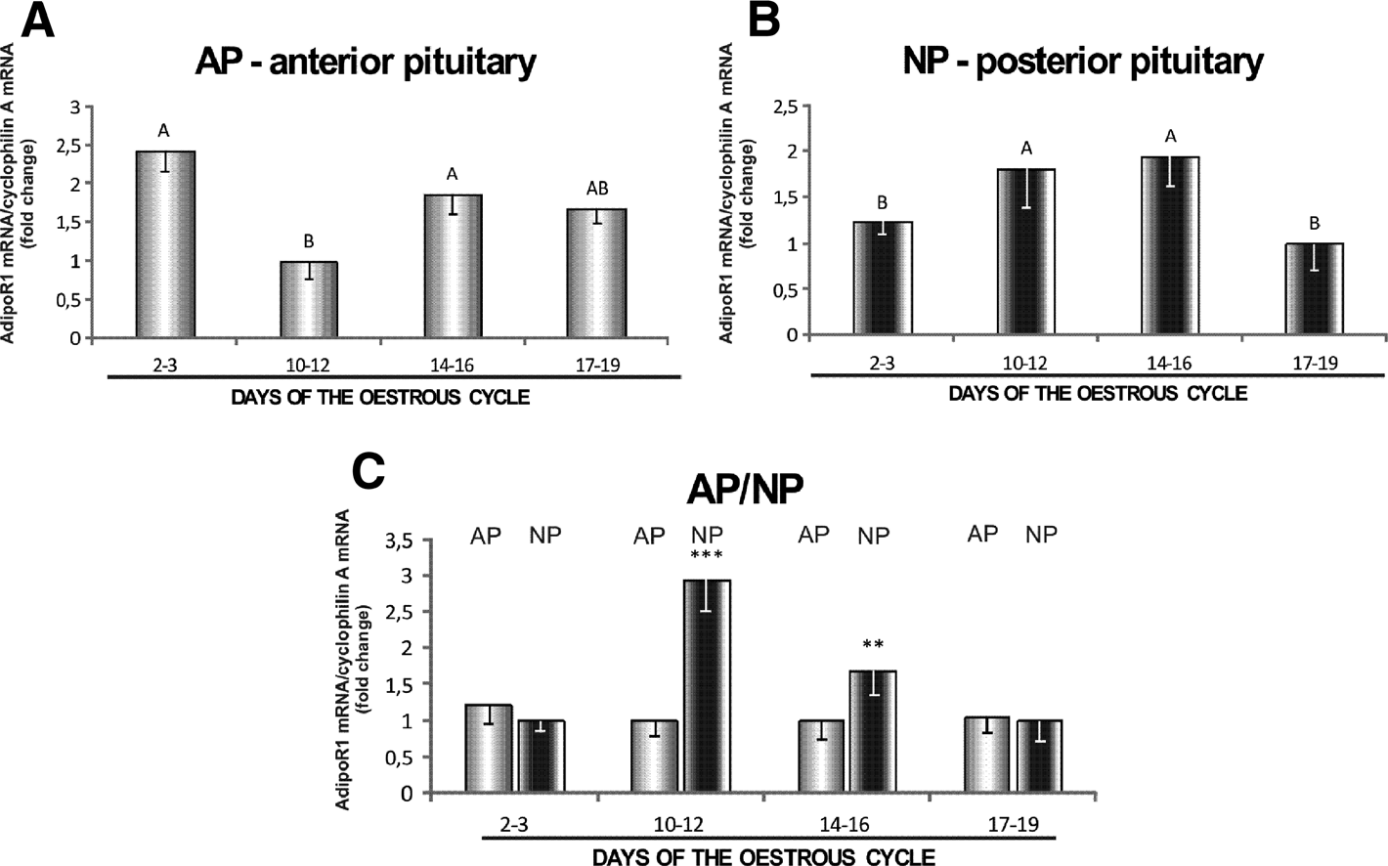
**Expression of AdipoR1 protein in the anterior and posterior pituitary.** A comparison of the expression levels of adiponectin receptor 1 (AdipoR1) protein determined by Western blotting analysis in porcine anterior (**A**) and posterior (**B**) pituitary glands between days 2–3, 10–12, 14–16 and 17–19 of the oestrous cycle, and (**C**) between anterior (AP) and posterior (NP) pituitary glands on days 2–3, 10–12, 14–16 and 17–19 of the cycle. Upper panels: representative immunoblots (MM, molecular marker; SM, skeletal muscles-positive control); lower panels: densitometric analysis of adiponectin receptor 1 protein relative to actin protein. Values are expressed as means ± S.E.M. in arbitrary optical density units (n = 5). Bars with different superscripts are significantly different. Capital letters indicate *p* < 0.05; ***p < 0.001.

**Figure 4:**
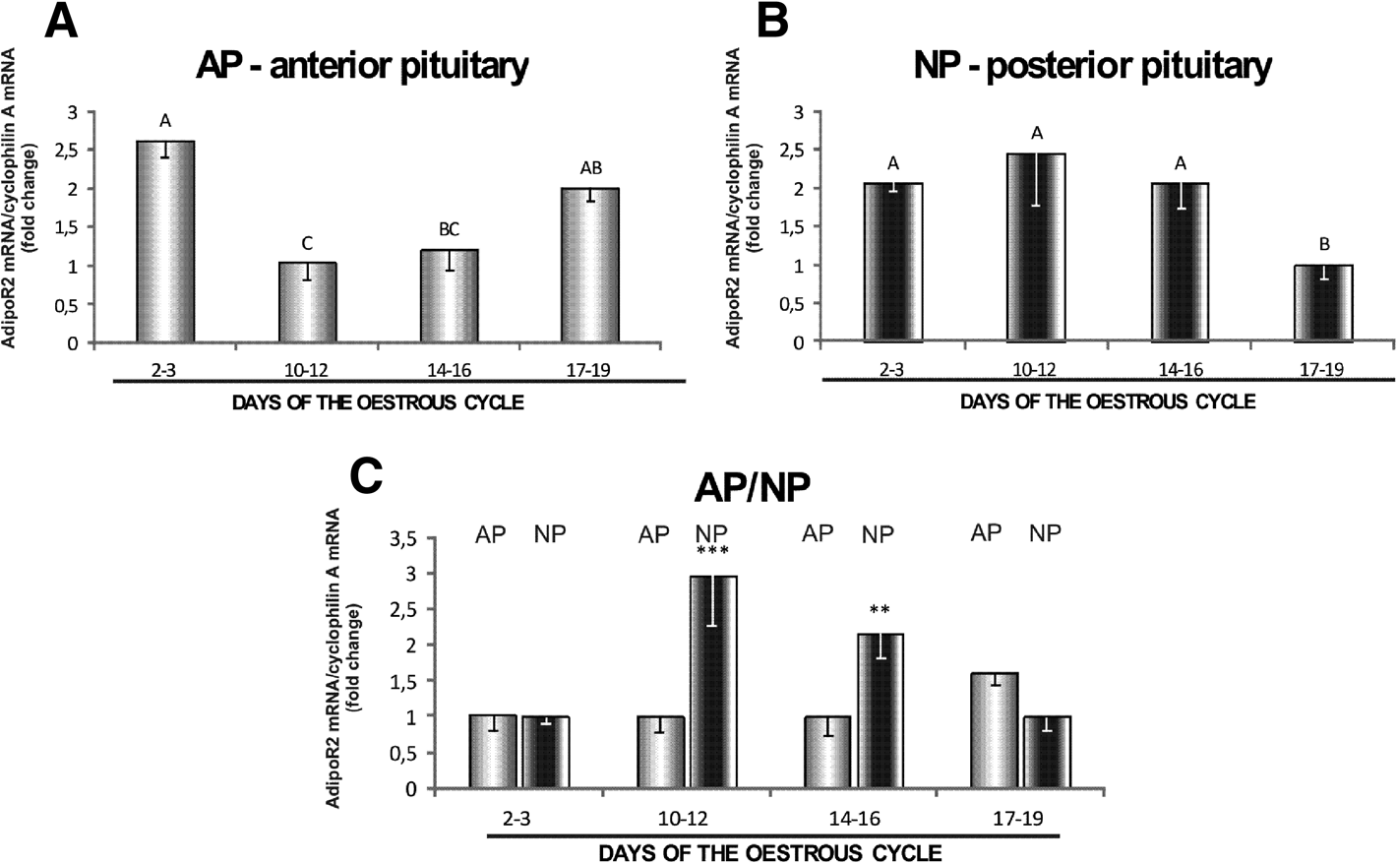
**Expression of AdipoR2 mRNA in the anterior and posterior pituitary.** Comparison of adiponectin receptor 2 (AdipoR2) protein expression determined by Western blotting analysis in porcine anterior (**A**) and posterior (**B**) pituitary glands between days 2–3, 10–12, 14–16 and 17–19 of the oestrous cycle, and (**C**) between anterior and posterior pituitary glands on days 2–3, 10–12, 14–16 and 17–19 of the cycle. Upper panels: representative immunoblots (MM, molecular marker; L, liver-positive control); lower panels: densitometric analysis of adiponectin receptor 2 protein relative to actin protein. Values are expressed as means ± S.E.M. in arbitrary optical density units (n = 5). Bars with different superscripts are significantly different. Capital letters indicate *p* < 0.05; **p < 0.01; ***p < 0.001.

AdipoR1 protein levels in AP were lowest on days 17–19 (*p* < 0.05), and no differences were observed between the remaining periods (Figure 3A). In NP, the variations in protein expression during the oestrous cycle were negligible (Figure 3B). AdipoR1 protein concentrations were higher in AP than in NP on days 2–3, 10–12 and 14–16 (*p* < 0.05) of the oestrous cycle (Figure 3C). AdipoR2 protein in AP was most abundant on days 10–12, and its levels were lowest on days 2–3 and 17–19 of the cycle (*p* < 0.05) (Figure 4A). The presence of AdipoR2 protein in NP was more pronounced on days 10–12, and it reached the lowest level on days 2–3 (*p* < 0.05) (Figure 4B). AdipoR2 protein was also more abundant in AP than in NP on days 14–16 (*p* < 0.05), and higher levels of expression were noted in NP than in AP on days 17–19 (*p* < 0.05) of the oestrous cycle (Figure 4C).

## Discussion

Our study was the first experiment to demonstrate the level of AdipoR1 and AdipoR2 genes transcripts and proteins in both anterior and posterior pituitaries of cyclic gilts. We found that the stage of the oestrous cycle affects the abundance of mRNAs and proteins of both adiponectin receptors. The abundance of AdipoR1 and AdipoR2 mRNAs varied throughout the oestrous cycle, with a marked decrease in AdipoR1 levels on days 10–12 in AP and an increase on days 10–12 and 14–16 in NP. By contrast, AdipoR2 transcript content was higher on days 2–3 and 17–19 in AP and during the entire luteal phase in NP. Unlike the level of the AdipoR1 mRNA, the content of AdipoR1 protein was elevated during the entire luteal phase in AP, but it did not differ in NP during the oestrous cycle. AdipoR2 protein concentrations in AP and NP reached the highest level on days 10–12 of the oestrous cycle. Indicated in the study, lack of correlation between the protein concentration and gene transcripts may result from transcriptional regulation, post-transcriptional regulation (RNA processing and stability), translation regulation or protein stability, as well as functioning feedbacks, i.e. high protein concentration may suppress mRNA expression, and a high level of gene expression may diminish the post-transcriptional processes. The low concentration of protein with the simultaneous high gene expression level can also be caused by the action of interference RNA (RNAi). The above suggests that tissue mRNA and protein levels are determined by physiological state, and they are not always correlated.

There is a scarcity of data regarding adiponectin system (adiponectin and adiponectin receptors) expression in the pituitary. The presence of mRNA for all system components has been reported only in male rats [20,21], humans [21] and chickens [16]. Adiponectin receptor genes are also expressed by somatotroph cells isolated from transgenic GFP expressing mice, the GH3 cell line (rat pituitary tumor cell line) and LβT2 immortalized mouse gonadotrophs [20,27]. The presence of adiponectin, AdipoR1 and AdipoR2 proteins in the human pituitary has been demonstrated by immunohistochemical methods. Interestingly, Psilopanagioti et al. [19] observed the colocalization of AdipoRs with gonadotrophs, somatotrophs and thyrotrophs, but not with corticotrophs or lactotrophs. Our study provides the first documented evidence of the expression of adiponectin receptors in the anterior and posterior lobes of the porcine pituitary during the oestrous cycle. The expression of the adiponectin in the studied endocrine gland was also determined (Kaminski et al., data not shown). The presence of both ligand and receptors in porcine pituitary may suggest adiponectin’s auto-/paracrine role in the regulation of the gland function.

The noted variations in the expression of AdipoRs during the cycle suggest a correlation with the animals’ hormonal milieu, primarily at the level of steroid hormones. Heightened levels of AdipoR1 and AdipoR2 mRNAs in the posterior lobe and lowered concentrations in the anterior lobe of the porcine pituitary during the luteal phase of the oestrous cycle could be attributed to ovarian hormones. The up-regulating effects of progesterone on AdipoRs transcripts in NP and its down-regulating effects in AP cannot be ruled out. This hypothesis seems to be confirmed by the results of Takemura et al. [28] who observed a similar pattern of AdipoRs mRNA expression in the human endometrium and attributed their findings to endometrial changes during the implantation period. In a study of rat placenta during gestation, Caminos et al. [29] noted that progesterone had a stimulating effect on AdipoR2 gene expression. In addition to progesterone, oestradiol could be yet another ovarian hormone to be involved in AdipoRs expression. Tabandeh et al. [30] postulated that enhanced expression of adiponectin receptors in theca, granulosa and cumulus cells of bovine ovarian follicles, especially in late stages of follicular growth, could result from increased oestradiol concentrations in follicular fluid. Lagaly et al. [31] reported a similar trend in AdipoR2 gene expression in theca cells from large follicles of beef cattle. Interestingly, Tan et al. [32] observed that oestradiol and testosterone increase AdipoRs mRNA and protein levels in cultured human adipocytes. An *in vitro* study of 3 T3-L1 cells (differentiated into adipocyte-like phenotype) revealed an increase in AdipoR1 and AdipoR2 gene transcript levels after oestradiol treatment [33]. It has also been suggested that other factors, like insulin, prolactin, growth hormone or cytokines, may influence the abundance of AdipoRs [34-36]. Interestingly, several authors have suggested that adiponectin could be an important factor in modulating its own receptor levels. In a study by Rodriguez-Pacheco et al. [21], a significant decrease in AdipoR1 mRNA levels and an increase in AdipoR2 mRNA concentrations was noted in cultures of rat pituitary cells exposed to 10^-8^ M and 10^-7^ M of adipokine for 24 h, respectively. Data contrary to the above findings were reported by Caminos et al. [29] who observed a suppressing effect of adiponectin on AdipoR2 mRNA expression in cultured human placenta explants. The above results strongly suggest that steroids and other factors, including adiponectin itself, may affect the expression of adiponectin receptors in the pituitary.

Hormonal regulation of the adiponectin system is not limited to the receptors, and the production of adiponectin is also modified by steroid and protein hormones. Böttner et al. [37] demonstrated that serum adiponectin levels change between childhood and adulthood in a negative correlation with serum androgen levels. Elevated levels of adipokine were also reported in patients with hypogonadotropic hypogonadism and anorexia nervosa, whereas the administration of testosterone decreased adiponectin concentrations [38-40]. A similar relationship was observed in mice [41]. The role of oestrogens remains unclear. Inhibitory effects of oestradiol on plasma adiponectin in women and female ovariectomized rats [42-44] as well as lack of any action [45,46] were both noted. Some data suggest that other factors, like prolactin, growth hormone, insulin or hCG also influence adiponectin expression [35,43,47].

The expression of both adiponectin receptors in the pituitary suggests that adiponectin has a local modulatory effect on central endocrine axes and that it participates in central control of metabolic homeostasis. *In vitro* studies seem to confirm this hypothesis. In a pituitary cell culture, short-term exposure (4 h) to the discussed hormone inhibited LH and GH release. Adiponectin reduced the stimulatory effect of GnRH on LH release, whereas GH release evoked by GHRH was not affected [21]. The above results were validated by Lu et al. [27] in whose study, adiponectin had an inhibitory effect on basal and GnRH-stimulated LH release, but not on FSH release by LβT2 cells. The discussed findings seem to confirm the hypothesis that adiponectin is involved in auto-/paracrine control of pituitary functions.

Adiponectin may also act at the hypothalamic level. The expression of all adiponectin system components was observed in porcine hypothalamic structures responsible for GnRH secretion: the medio-basal hypothalamus (MBH), preoptic area (POA) and median eminence (SME) (Kaminski et al., data not shown). Adiponectin receptors were also expressed in rat POA [48], as well as in the arcuate nucleus (ARH) and paraventricular hypothalamus (PVH) of mice [49]. The presence of AdipoRs was observed in magnocellular (oxytocin or vasopressin secreting) neurons of rat PVH [50]. Adiponectin’s influence on the excitability of oxytocin-secreting neurons could explain enhanced oxytocin secretion in an obese population [50]. Globular adiponectin significantly decreased GnRH release in GT1-7 cells *via* AMPK activation. Specifically, low levels of adiponectin may contribute to chronically elevated LH levels [51]. The discussed research findings indicate that adiponectin may be an important factor which regulates pituitary functions directly as well as by modulating hypothalamic activity.

## Conclusions

Our study was the first experiment to demonstrate the presence of AdipoR1 and AdipoR2 mRNAs and proteins in porcine pituitary and the effect of different stages of the oestrous cycle on the expression of both receptors. The presumable occurrence of AdipoRs in the pituitary suggests that adiponectin may affect reproductive functions by controlling the hypothalamic-pituitary-gonadal axis. Adiponectin’s role in the anterior and posterior pituitary is poorly understood, and further work is needed to investigate its functions in detail.

## Competing interests

The authors declare that they have no competing interests.

## Authors’ contributions

MK participated in design of the study, protein isolation, carried Western Blot analysis, prepared the statistical analysis and drafted the manuscript. AM participated in the mRNA and protein isolation, carried real-time analysis. AN collected the tissues, participated in mRNA and protein isolation. NS participated in the design of the study and coordination. TK conceived, designed and coordinated the study, helped to draft the manuscript. All authors read and approved the final manuscript.
